# Myeloid-Derived Vascular Endothelial Growth Factor and Hypoxia-Inducible Factor Are Dispensable for Ocular Neovascularization—Brief Report

**DOI:** 10.1161/ATVBAHA.115.306681

**Published:** 2015-12-23

**Authors:** Sidath E. Liyanage, Alessandro Fantin, Pilar Villacampa, Clemens A. Lange, Laura Denti, Enrico Cristante, Alexander J. Smith, Robin R. Ali, Ulrich F. Luhmann, James W. Bainbridge, Christiana Ruhrberg

**Affiliations:** From the Divisions of Genetics (S.E.L., P.V., C.A.L., E.C., A.J.S., R.R.A., U.F.L., J.W.B.) and Cell Biology (A.F., L.D., C.R.), UCL Institute of Ophthalmology, University College London, London, United Kingdom.

**Keywords:** choroidal neovascularization, diabetic retinopathy, macular degeneration, myeloid cells, vascular endothelial growth factor A

## Abstract

Ocular neovascularization (ONV) is a pathological feature of sight-threatening human diseases, such as diabetic retinopathy and age-related macular degeneration. Macrophage depletion in mouse models of ONV reduces the formation of pathological blood vessels, and myeloid cells are widely considered an important source of the vascular endothelial growth factor A (VEGF). However, the importance of VEGF or its upstream regulators hypoxia-inducible factor-1α (HIF1α) and hypoxia-inducible factor-2α (HIF2α) as myeloid-derived regulators of ONV remains to be determined.

**Approach and Results—:**

We used 2 mouse models of ONV, choroidal neovascularization and oxygen-induced retinopathy, to show that *Vegfa* is highly expressed by several cell types, but not myeloid cells during ONV. Moreover, myeloid-specific VEGF ablation did not reduce total ocular VEGF during choroidal neovascularization or oxygen-induced retinopathy. In agreement, the conditional inactivation of *Vegfa*, *Hif1a*, or *Epas1* in recruited and resident myeloid cells that accumulated at sites of neovascularization did not significantly reduce choroidal neovascularization or oxygen-induced retinopathy.

**Conclusions—:**

The finding that myeloid cells are not a significant local source of VEGF in these rodent models of ONV suggests that myeloid function in neovascular eye disease differs from skin wound healing and other neovascular pathologies.

## Abstract 2

Supplemental Digital Content is available in the text.

Myeloid-derived vascular endothelial growth factor (VEGF) has been proposed to drive ocular neovascularization (ONV),^1–4^ a pathological feature common to leading causes of blindness, including retinopathy of prematurity in infants, proliferative diabetic retinopathy in the working population, and age-related macular degeneration in the elderly.^5^ In mice with oxygen-induced retinopathy (OIR), a model of retinopathy of prematurity, VEGF-expressing macrophages are recruited to sites of retinal neovascularization (RNV), and clodronate-induced or genetic macrophage depletion reduces RNV, raising the possibility that myeloid-derived VEGF promotes RNV.^3,6,7^ In laser-induced choroidal neovascularization (CNV), a mouse model of age-related macular degeneration–associated neovascularization, peak VEGF expression correlates with maximal myeloid infiltration, and clodronate-induced macrophage depletion reduces both VEGF levels and CNV area.^1^ The absence of VEGF-producing CCR2^+^ macrophages also reduces CNV area.^2^ Human CNV lesions have also been reported to contain VEGF-expressing macrophages, which were suggested to cooperate with VEGF-expressing retinal pigment epithelium (RPE) to drive angiogenesis.^8^ These findings raised the possibility that myeloid-derived VEGF also promotes CNV. However, others contested that myeloid-derived VEGF enhances CNV.^9^ The significance of myeloid-derived VEGF in ONV, therefore, remains controversial. Moreover, the importance of myeloid-derived hypoxia-inducible factors, HIF1α and HIF2α, has not yet been defined for ONV, even though they regulate VEGF expression,^10^ have been implicated in myeloid-mediated angiogenesis in various tissues^11^ and are expressed in OIR and CNV models.^12,13^ To test the prevailing idea in the current literature that myeloid VEGF is nonredundant with other VEGF sources in ONV, we used conditional mouse knockout models to target *Vegfa* and its upstream regulators, *Hif1a* and *Epas1* (*Hif2a*), in myeloid cells, and analyzed the effects of their deletion on RNV and CNV. Unexpectedly, we found that myeloid-derived HIFs and VEGF are dispensable ONV, suggesting that they do not present useful targets for therapy of ocular disease.

**See cover image**

## Materials and Methods

Materials and Methods are available in the online-only Data Supplement.

Briefly, animal procedures were conducted with ethical approval under institutional and UK Home Office guidelines using *Lysm*^*+/Cre*^*;Hif1a*^*fl/fl*^, *Lysm*^*+/Cre*^*;Epas1*^*fl/fl*^ and *Lysm*^*+/Cre*^;*Vegfa*^*fl/fl*^^11,14,15^, *Tie2-Cre;Vegfa*^*fl/fl*^,^16^ *Vegfa*^*+/LacZ*^, *Rosa26*^*Yfp*^, and *Rosa26*^*mT/mG*^^17–19^ mice in OIR^20^ and CNV^21^ assays for gene expression, *Cre* recombination and ONV assays.

## Results

**Figure 1.**
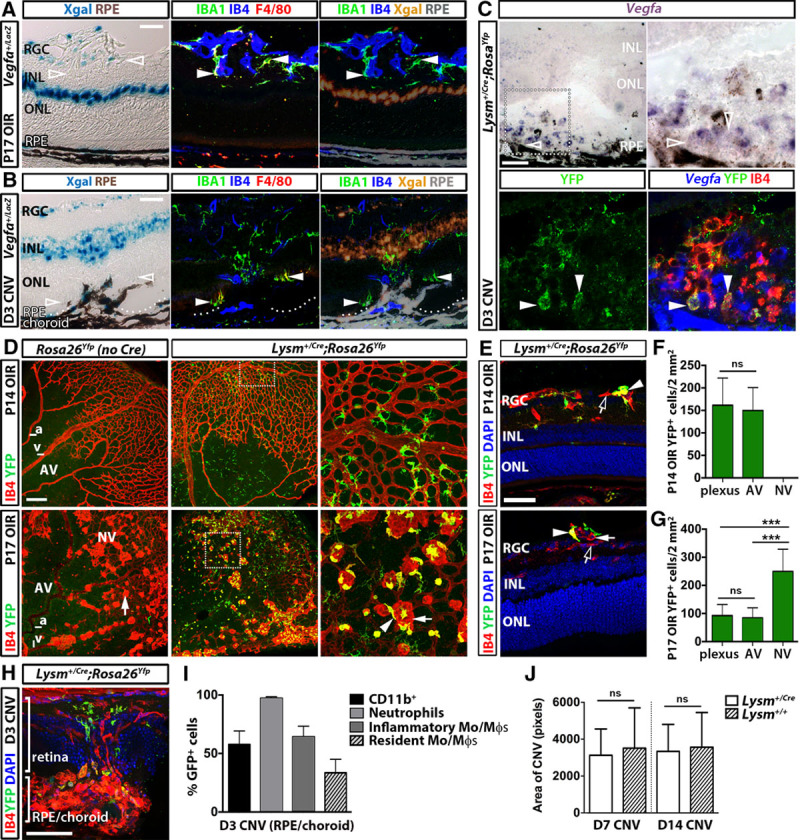
Myeloid cells accumulate at sites of ocular neovascularization (ONV), but are not a significant source of *Vegfa*. **A**–**C**, *Vegfa* expression in ONV. X-gal staining (**left**) followed by labeling for IBA1, F4/80, and IB4 (**middle**) of *Vegfa*^*+/LacZ*^ eyes on P17 in the oxygen-induced retinopathy (OIR) model (**A**) or on D3 after laser injury (**B**). X-gal was pseudocolored orange and retinal pigment epithelium (RPE) pigment gray for overlay with fluorescent signals (**right**). *Vegfa* in situ hybridization (**C**) of an *Lysm*^*+/Cre*^;*Rosa26*^*Yfp*^ eye section on D3 after laser injury, shown at higher magnification on the **right**. **Bottom**, **C**, The *Vegfa* signal was inverted into the blue channel for overlay with YFP and IB4 staining. Arrowheads indicate examples of IB4^+^ YFP^+^ myeloid cells and clear arrowheads indicate their lack of *Vegfa* expression. **D** and **E**, Retinal flatmounts (**D**) and sections (**E**) of *Lysm*^*+/Cre*^*;Rosa26*^*Yfp*^ OIR retinas labeled for IB4 and YFP on P14 (**top**) or P17 (**bottom**), counterstaind with 4′,6-diamidino-2-phenylindole (DAPI). Examples of quiescent vessels (clear arrows) and YFP^+^ IB4^+^ myeloid cells (arrowheads) associated with neovascular tufts (arrows) are indicated. Areas indicated by squares are shown at higher magnification in adjacent panels (**D**). **F** and **G**, Quantification of YFP^+^ cells in the vascular plexus, avascular (AV) and neovascular (NV) areas of *Lysm*^*+/Cre*^*; Rosa26*^*Yfp*^ retinal flatmounts on P14 (**F**) and P17 (**G**) in the OIR model; n≥5 mice each, ***P<0.001 for NV vs AV or vascular plexus, 1-way ANOVA. **H**, YFP^+^ myeloid cells in *Lysm*^*+/Cre*^*;Rosa26*^*Yfp*^ adult eye sections on D3 after laser injury. **I**, Flow cytometric analysis of the choroid/RPE shows reporter activation in CD11b^+^ myeloid cells and myeloid subsets in *Lysm*^*+/Cre*^*;Rosa26*^*mT/mG*^ eyes on D3 after laser injury; n≥5 each. **J**, Similar lesion area in *Lysm*^*+/Cre*^ and *Lysm*^*+/+*^ mice after laser injury; n≥11 mice each, *P*>0.05, *t* test. a indicates artery; CNV, choroidal neovascularization; INL, inner nuclear layer; ns, not significant; ONL, outer nuclear layer; RGC, retinal ganglion cell layer; and v, vein. Scale bars, 50 μm (**A**, **B**, **C**, **E**, and **H**), 200 μm (**D**).

We induced OIR or CNV in *Vegfa*^*+/LacZ*^ mice, previously shown to faithfully report *Vegfa* gene expression in macrophages and other cells types.^17,22^ X-gal staining of eye sections indicated prominent *Vegfa* expression in the RPE, inner nuclear layer, and retinal ganglion cell layer on postnatal day (P) 17 in the OIR and on day (D) 3 postlasering in the CNV model, when VEGF levels and myeloid infiltration peak,^1,23^ but *Vegfa* expression was below the detection limit in IBA1^+^ F4/80^+^ microglia/macrophages (Figure 1A and 1B). *Vegfa* expression was also undetectable in YFP^+^ IB4^+^ myeloid cells by in situ hybridization on D3 after lasering *Lysm*^*+/Cre*^ eyes carrying the *Rosa26*^*Yfp*^ reporter to identify myeloid cells, even though other cell types strongly expressed *Vegfa* (Figure 1C). These findings suggest that, compared with other ocular cell types, myeloid cells are unlikely a significant local source of VEGF for ONV.

OIR retinas from *Lysm*^*+/Cre*^*;Rosa26*^*Yfp*^ myeloid reporter mice accumulated YFP^+^ myeloid cells in both avascular and vascularized areas at P14, before the onset of RNV (Figure 1D–1F). By P17, YFP^+^ myeloid cells had accumulated near neovascular tufts (Figure 1D, 1E, and 1G). We also observed YFP-expressing myeloid cells at sites of laser injury in *Lysm*^*+/Cre*^*;Rosa26*^*Yfp*^ eyes on D3 postlasering, the onset of CNV (Figure 1H). Flow cytometry analysis of D3 *Lysm*^*+/Cre*^*;Rosa26*^*mT/mG*^ choroid/RPE complex showed efficient recombination in infiltrating CD11b^+^ myeloid cells, particularly neutrophils and inflammatory monocytes/macrophages (Figure 1I). Importantly, the *Lysm*^*Cre*^ allele did not affect the size of avascular or neovascular areas on P17 (*Lysm^+/Cre^*: 6.9±1.9%; *Lysm^+/+^*: 7.6±2.8%; mean±SD, n=15, *P*>0.05) or CNV lesions on D7 or D14 postlasering (Figure 1J). *Lysm*^*+/Cre*^ is therefore a suitable tool to target genes in myeloid cells recruited to sites of ONV.

**Figure 2.**
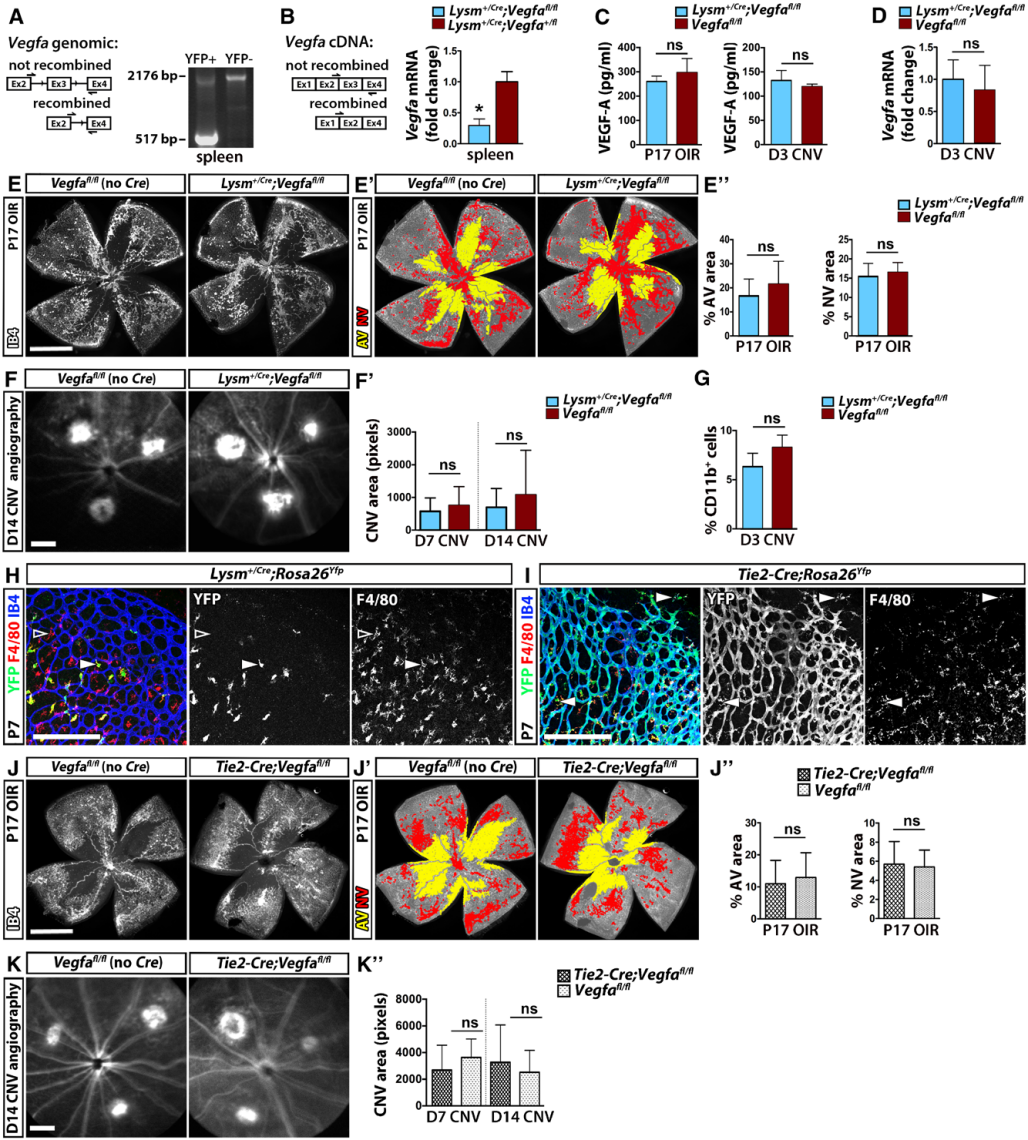
Myeloid-derived *Vegfa* does not significantly contribute to the total vascular endothelial growth factor (VEGF) pool or ocular neovascularization. (**A** and **B**) Polymerase chain reaction detection of *Vegfa* gene (**A**) and mRNA recombination (**B**) in YFP^+^ splenocytes in *Vegfa*^*fl/fl*^;*Lysm*^*+/Cre*^;*Rosa26*^*Yfp*^ mutants (**A** and **B**) and *Vegfa*^*+/fl*^;*Lysm^+/Cre^;Rosa26^Yfp^* controls (**B**); n≥3 mice each, *P*<0.05, *t* test. (**C** and **D**) VEGF protein levels (**C**) in the P17 oxygen-induced retinopathy (OIR) retina (**left**) and the retinal pigment epithelium (RPE)/choroid on D3 after laser injury (**right**) and *Vegfa* mRNA (mean fold change relative to *Actb*, (**D**) in the RPE/choroid on D3 after laser injury in *Lysm*^*+/Cre*^;*Vegfa*^*fl/fl*^ mice and control littermates; mean±SD, n≥3 each; *P*>0.05, *t* test. (**E**–**E″**) IB4 staining (**E**) of P17 OIR *Lysm*^*+/Cre*^*;Vegfa*^*fl/fl*^ and control retina. **E′**, Total retina, avascular (AV) and neovascular (NV) areas are rendered gray, yellow, and red, respectively. **E″**, Proportion of central AV and NV areas; mean±SD, n≥6 each; *P*>0.05, *t* test. **F**–**G**, D14 angiograms (**F**) and choroidal neovascularization (CNV) lesion area on D7 and D14 (**F′**) and percentage of CD11b^+^ cells in choroid/RPE on D3 after laser injury (**G**) of *Lysm*^*+/Cre*^*;Vegfa*^*fl/fl*^ and control mice; mean±SD, n≥4 each; *P*>0.05, *t* test. **H** and **I**, Wholemount retina staining for IB4, F4/80, and YFP shows recombination in microglia in *Lysm*^*+/Cre*^*;Rosa*^*Yfp*^ mice (**H**) and in most microglia and endothelium in *Tie2-Cre;Rosa*^*Yfp*^ mice (**I**). **J**–**J″**, IB4 staining (**J**) of P17 OIR *Tie2-Cre;Vegfa*^*fl/fl*^ and control retina. **J′**, Total retina, AV, and NV areas are rendered gray, yellow, and red, respectively. **J″**, Proportion of central AV and NV areas in *Tie2-Cre;Vegfa*^*fl/fl*^ and control P17 OIR retina stained with IB4; mean±SD, n≥5 mice each; *P*>0.05, *t* test. **K and K′**, D14 angiograms (**K**) and quantification of CNV lesion area on D7 and D14 after laser injury (**K′**) in *Tie2-Cre;Vegfa*^*fl/fl*^ mice and littermate controls; n≥5 mice each, *P*>0.05, *t* test. Scale bars, 1 mm (**E**, **F**, **J**, and **K**), 200 μm (**H** and **I**).

Next, we examined *Lysm*^*+/Cre*^*;Vegfa*^*fl/fl*^ mice, which are deficient in myeloid cell–derived *Vegfa* and were previously shown to have reduced pathological angiogenesis in wound healing and cancer models.^22,24^ *Lysm*^*+/Cre*^*;Vegfa*^*fl/fl*^ mice appeared healthy as previously reported and had normal retinal angiogenesis (Figure IA in the online-only Data Supplement). YFP-expressing splenic myeloid cells showed efficient *Vegfa* gene targeting and, accordingly, *Vegfa* mRNA was reduced in mutant compared with control YFP^+^ splenic myeloid cells (Figure 2A and 2B). Nevertheless, myeloid VEGF deletion did not alter overall VEGF protein or mRNA levels in the P17 OIR retina or D3 postlasering RPE/choroid (Figure 2C and 2D). In agreement, the size of the central avascular and neovascular areas in P17 OIR retina and D7 and D14 CNV lesions was similar in *Lysm*^*+/Cre*^*;Vegfa*^*fl/fl*^ mice and controls (Figure 2E–2F′). Moreover, myeloid VEGF depletion did not affect CD11b^+^ cell recruitment to the RPE/choroid on D3 postlasering (Figure 2G).

We also examined *Tie2-Cre;Vegfa*^*fl/fl*^ mice because *Tie2-Cre* targets yolk sac–derived tissue-resident macrophages more efficiently than *Lysm*^*Cre/+*^, including microglia in the brain^25,26^ and retina (Figure 2H and 2I). *Tie2-Cre;Vegfa*^*fl/fl*^ mutant mice are healthy, and despite targeting of *Vegfa* in hematopoietic and endothelial cells, have no obvious vascular defects and only develop vascular dysfunction in old age.^16,27^ In agreement, angiogenesis and the density of resident myeloid cells were similar in mutant and control postnatal retinas (Figure IB and IC in the online-only Data Supplement). Moreover, the size of the central avascular and neovascular areas in P17 OIR retina and CNV lesions was not significantly different between mutants and controls (Figure 2J–2K′). These data suggest that VEGF expression by resident microglia/macrophages does not explain the lack of angiogenesis defects in mice with *Lysm*^*Cre*^-mediated targeting of VEGF in myeloid cells. Myeloid cell–derived VEGF is therefore dispensable for retinal angiogenesis and pathological ONV.

Because HIFs promote the expression of *Vegfa* and other hypoxia-induced proangiogenic molecules,^10^ we also targeted the genes encoding HIF1α and HIF2α in myeloid cells with *Lysm*^*Cre*^. Targeting of *Hif1a*, *Epas1*, or both did not affect retinal vascular development, despite efficient *Lysm*^*+/Cre*^-mediated *Hif1a* or *Epas1* deletion in myeloid cells (Figure IIA and IIB in the online-only Data Supplement). Moreover, the size of the central avascular and neovascular areas on P17 after OIR (Figure IIIA and IIIB in the online-only Data Supplement) and D7 and D14 CNV lesions (Figure IIIC and IIID in the online-only Data Supplement) were similar in controls and mutants for *Hif1a*, *Epas1*, or both. The recruitment of myeloid cells, including individual subpopulations, to ONV sites was also not impaired after *Lysm*^*Cre*^-mediated targeting of *Hif1a*, *Epas1*, or both (Figure IIIE and IIIE′ in the online-only Data Supplement).

## Discussion

Nonmyeloid VEGF is thought to promote RNV because retinal ganglion cells^28,29^ and Mueller cells^30–32^ are abundant VEGF sources in the OIR model. Moreover, it was shown that the deletion of Mueller cell–derived VEGF in a mouse model of diabetes mellitus reduces RNV.^33^ Furthermore, RPE-derived VEGF has been implicated in CNV in both mice^34–36^ and patients,^8^ and HIF1α depletion in RPE cells impairs VEGF expression and reduces CNV in mice.^36,37^ VEGF expression and myeloid cell depletion studies have been interpreted as evidence that myeloid-derived VEGF provides an additional, nonredundant source of VEGF for both RNV and CNV.^1–4,8^ However, our studies show that myeloid expression of VEGF or its upstream regulators, HIF1α and HIF2α, is not necessary for ONV in rodent models of OIR and CNV. Previous studies deducing a role for myeloid-derived VEGF in ONV by correlating the phenotype caused by myeloid cell depletion with changes in VEGF levels^1–3^ may, therefore, have only identified an indirect association of both pathological parameters in eye disease. For example, myeloid cells may influence ONV indirectly by stimulating VEGF production by other cell types, such as the neural or glial sources previously implicated in ONV. Myeloid cells have also been found to influence angiogenesis by VEGF-independent mechanisms, for example, by acting as cellular chaperones to promote endothelial tip cell fusion during vascular development^26^ or by producing proangiogenic factors different from VEGF during tumor vascularization.^38^ The molecular mechanisms of inflammatory cell modulation of neovascular eye disease, therefore, differs significantly from nonocular disease models, in which myeloid-derived VEGF is nonredundant with other VEGF sources to promote pathological angiogenesis, even when nonmyeloid VEGF is abundant, for example, during tumor vascularization or in skin wound healing.^22,24^

## Acknowledgments

We thank Andy Joyce, Laura Abelleira, and the Biological Resources Unit staff at the UCL Institute of Ophthalmology for help with mouse husbandry and the Imaging Facility of the UCL Institute of Ophthalmology for maintenance of the confocal microscopes.

## Sources of Funding

This study was supported by a joint Medical Research Council and Fight for Sight grant (MR/K003003/1) to S.E. Liyanage, the People Programme (Marie Curie Actions) and the European Union’s Seventh Framework Programme (FP7/2007–2013) under Research Executive Agency grant agreement 629556 to P. Villacampa, the Special Trustees of Moorfields Eye Hospital (Grant ST 1503B) to J.W. Bainbridge and funding from the Wellcome Trust (095623/Z/11/Z) to C. Ruhrberg; J.W. Bainbridge is a Research Professor of the National Institute of Health Research.

## Disclosures

U.F. Luhmann is an employee of F. Hoffmann-La Roche Ltd. The other authors report no conflicts.

## Supplementary Material

## Significance

Previous work inferred from correlative studies that myeloid-derived vascular endothelial growth factor drives ocular neovascularization via induction of angiogenesis. Unexpectedly, we find that *Vegfa* is not expressed at significant levels by myeloid cells in the eye, and, accordingly, myeloid-derived vascular endothelial growth factor and its upstream regulator hypoxia-inducible factors are not required for ocular neovascularization. Our work implies organ-specific mechanisms by which myeloid cells regulate angiogenesis because myeloid cells do provide a significant and nonredundant source of vascular endothelial growth factor to promote pathological angiogenesis in other settings, such as skin wound healing and cancer. Moreover, our work suggests that understanding the role of myeloid cells in ocular angiogenesis requires focus on pathways unrelated to vascular endothelial growth factor or hypoxia-inducible factors.

## References

[1] Sakurai E, Anand A, Ambati BK, van Rooijen N, Ambati J (2003). Macrophage depletion inhibits experimental choroidal neovascularization.. Invest Ophthalmol Vis Sci.

[2] Krause TA, Alex AF, Engel DR, Kurts C, Eter N (2014). VEGF-production by CCR2-dependent macrophages contributes to laser-induced choroidal neovascularization.. PLoS One.

[3] Kataoka K, Nishiguchi KM, Kaneko H, van Rooijen N, Kachi S, Terasaki H (2011). The roles of vitreal macrophages and circulating leukocytes in retinal neovascularization.. Invest Ophthalmol Vis Sci.

[4] Liu J, Copland DA, Horie S, Wu WK, Chen M, Xu Y, Paul Morgan B, Mack M, Xu H, Nicholson LB, Dick AD (2013). Myeloid cells expressing VEGF and arginase-1 following uptake of damaged retinal pigment epithelium suggests potential mechanism that drives the onset of choroidal angiogenesis in mice.. PLoS One.

[5] Foster A, Resnikoff S (2005). The impact of Vision 2020 on global blindness.. Eye (Lond).

[6] Naug HL, Browning J, Gole GA, Gobé G (2000). Vitreal macrophages express vascular endothelial growth factor in oxygen-induced retinopathy.. Clin Experiment Ophthalmol.

[7] Kubota Y, Takubo K, Shimizu T, Ohno H, Kishi K, Shibuya M, Saya H, Suda T (2009). M-CSF inhibition selectively targets pathological angiogenesis and lymphangiogenesis.. J Exp Med.

[8] Grossniklaus HE, Ling JX, Wallace TM, Dithmar S, Lawson DH, Cohen C, Elner VM, Elner SG, Sternberg P (2002). Macrophage and retinal pigment epithelium expression of angiogenic cytokines in choroidal neovascularization.. Mol Vis.

[9] He L, Marneros AG (2013). Macrophages are essential for the early wound healing response and the formation of a fibrovascular scar.. Am J Pathol.

[10] Keith B, Johnson RS, Simon MC (2012). HIF1α and HIF2α: sibling rivalry in hypoxic tumour growth and progression.. Nat Rev Cancer.

[11] Cramer T, Yamanishi Y, Clausen BE, Förster I, Pawlinski R, Mackman N, Haase VH, Jaenisch R, Corr M, Nizet V, Firestein GS, Gerber HP, Ferrara N, Johnson RS (2003). HIF-1alpha is essential for myeloid cell-mediated inflammation.. Cell.

[12] Skeie JM, Mullins RF (2009). Macrophages in neovascular age-related macular degeneration: friends or foes?. Eye (Lond).

[13] Mowat FM, Luhmann UF, Smith AJ, Lange C, Duran Y, Harten S, Shukla D, Maxwell PH, Ali RR, Bainbridge JW (2010). HIF-1alpha and HIF-2alpha are differentially activated in distinct cell populations in retinal ischaemia.. PLoS One.

[14] Clausen BE, Burkhardt C, Reith W, Renkawitz R, Förster I (1999). Conditional gene targeting in macrophages and granulocytes using LysMcre mice.. Transgenic Res.

[15] Imtiyaz HZ, Williams EP, Hickey MM, Patel SA, Durham AC, Yuan LJ, Hammond R, Gimotty PA, Keith B, Simon MC (2010). Hypoxia-inducible factor 2alpha regulates macrophage function in mouse models of acute and tumor inflammation.. J Clin Invest.

[16] Cattin AL, Burden JJ, Van Emmenis L (2015). Macrophage-induced blood vessels guide Schwann cell-mediated regeneration of peripheral nerves.. Cell.

[17] Miquerol L, Gertsenstein M, Harpal K, Rossant J, Nagy A (1999). Multiple developmental roles of VEGF suggested by a LacZ-tagged allele.. Dev Biol.

[18] Srinivas S, Watanabe T, Lin CS, William CM, Tanabe Y, Jessell TM, Costantini F (2001). Cre reporter strains produced by targeted insertion of EYFP and ECFP into the ROSA26 locus.. BMC Dev Biol.

[19] Muzumdar MD, Tasic B, Miyamichi K, Li L, Luo L (2007). A global double-fluorescent Cre reporter mouse.. Genesis.

[20] Smith LE, Wesolowski E, McLellan A, Kostyk SK, D’Amato R, Sullivan R, D’Amore PA (1994). Oxygen-induced retinopathy in the mouse.. Invest Ophthalmol Vis Sci.

[21] Balaggan KS, Binley K, Esapa M, MacLaren RE, Iqball S, Duran Y, Pearson RA, Kan O, Barker SE, Smith AJ, Bainbridge JW, Naylor S, Ali RR (2006). EIAV vector-mediated delivery of endostatin or angiostatin inhibits angiogenesis and vascular hyperpermeability in experimental CNV.. Gene Ther.

[22] Willenborg S, Lucas T, van Loo G, Knipper JA, Krieg T, Haase I, Brachvogel B, Hammerschmidt M, Nagy A, Ferrara N, Pasparakis M, Eming SA (2012). CCR2 recruits an inflammatory macrophage subpopulation critical for angiogenesis in tissue repair.. Blood.

[23] Eter N, Engel DR, Meyer L, Helb HM, Roth F, Maurer J, Holz FG, Kurts C (2008). *In vivo* visualization of dendritic cells, macrophages, and microglial cells responding to laser-induced damage in the fundus of the eye.. Invest Ophthalmol Vis Sci.

[24] Stockmann C, Doedens A, Weidemann A, Zhang N, Takeda N, Greenberg JI, Cheresh DA, Johnson RS (2008). Deletion of vascular endothelial growth factor in myeloid cells accelerates tumorigenesis.. Nature.

[25] Tang Y, Harrington A, Yang X, Friesel RE, Liaw L (2010). The contribution of the Tie2+ lineage to primitive and definitive hematopoietic cells.. Genesis.

[26] Fantin A, Vieira JM, Gestri G, Denti L, Schwarz Q, Prykhozhij S, Peri F, Wilson SW, Ruhrberg C (2010). Tissue macrophages act as cellular chaperones for vascular anastomosis downstream of VEGF-mediated endothelial tip cell induction.. Blood.

[27] Lee S, Chen TT, Barber CL, Jordan MC, Murdock J, Desai S, Ferrara N, Nagy A, Roos KP, Iruela-Arispe ML (2007). Autocrine VEGF signaling is required for vascular homeostasis.. Cell.

[28] Stone J, Chan-Ling T, Pe’er J, Itin A, Gnessin H, Keshet E (1996). Roles of vascular endothelial growth factor and astrocyte degeneration in the genesis of retinopathy of prematurity.. Invest Ophthalmol Vis Sci.

[29] Sapieha P, Sirinyan M, Hamel D (2008). The succinate receptor GPR91 in neurons has a major role in retinal angiogenesis.. Nat Med.

[30] Eichler W, Kuhrt H, Hoffmann S, Wiedemann P, Reichenbach A (2000). VEGF release by retinal glia depends on both oxygen and glucose supply.. Neuroreport.

[31] Pierce EA, Avery RL, Foley ED, Aiello LP, Smith LE (1995). Vascular endothelial growth factor/vascular permeability factor expression in a mouse model of retinal neovascularization.. Proc Natl Acad Sci U S A.

[32] Rodrigues M, Xin X, Jee K, Babapoor-Farrokhran S, Kashiwabuchi F, Ma T, Bhutto I, Hassan SJ, Daoud Y, Baranano D, Solomon S, Lutty G, Semenza GL, Montaner S, Sodhi A (2013). VEGF secreted by hypoxic Müller cells induces MMP-2 expression and activity in endothelial cells to promote retinal neovascularization in proliferative diabetic retinopathy.. Diabetes.

[33] Wang J, Xu X, Elliott MH, Zhu M, Le YZ (2010). Müller cell-derived VEGF is essential for diabetes-induced retinal inflammation and vascular leakage.. Diabetes.

[34] Wada M, Ogata N, Otsuji T, Uyama M (1999). Expression of vascular endothelial growth factor and its receptor (KDR/flk-1) mRNA in experimental choroidal neovascularization.. Curr Eye Res.

[35] Julien S, Kreppel F, Beck S, Heiduschka P, Brito V, Schnichels S, Kochanek S, Schraermeyer U (2008). A reproducible and quantifiable model of choroidal neovascularization induced by VEGF A165 after subretinal adenoviral gene transfer in the rabbit.. Mol Vis.

[36] Kurihara T, Westenskow PD, Bravo S, Aguilar E, Friedlander M (2012). Targeted deletion of Vegfa in adult mice induces vision loss.. J Clin Invest.

[37] Lin M, Hu Y, Chen Y, Zhou KK, Jin J, Zhu M, Le YZ, Ge J, Ma JX (2012). Impacts of hypoxia-inducible factor-1 knockout in the retinal pigment epithelium on choroidal neovascularization.. Invest Ophthalmol Vis Sci.

[38] Murdoch C, Muthana M, Coffelt SB, Lewis CE (2008). The role of myeloid cells in the promotion of tumour angiogenesis.. Nat Rev Cancer.

