# Supplementary material for: Myeloid-Derived Vascular Endothelial Growth Factor and Hypoxia-Inducible Factor Are Dispensable for Ocular Neovascularization—Brief Report
Source: Arterioscler Thromb Vasc Biol. 2015 Dec 23;36(1):19–24. doi: 10.1161/ATVBAHA.115.306681 (PMC4684248; doi:10.1161/ATVBAHA.115.306681)
Supplement: Supplementary file 1 [file atv-36-19-s001.pdf]

Supplemental Figure I

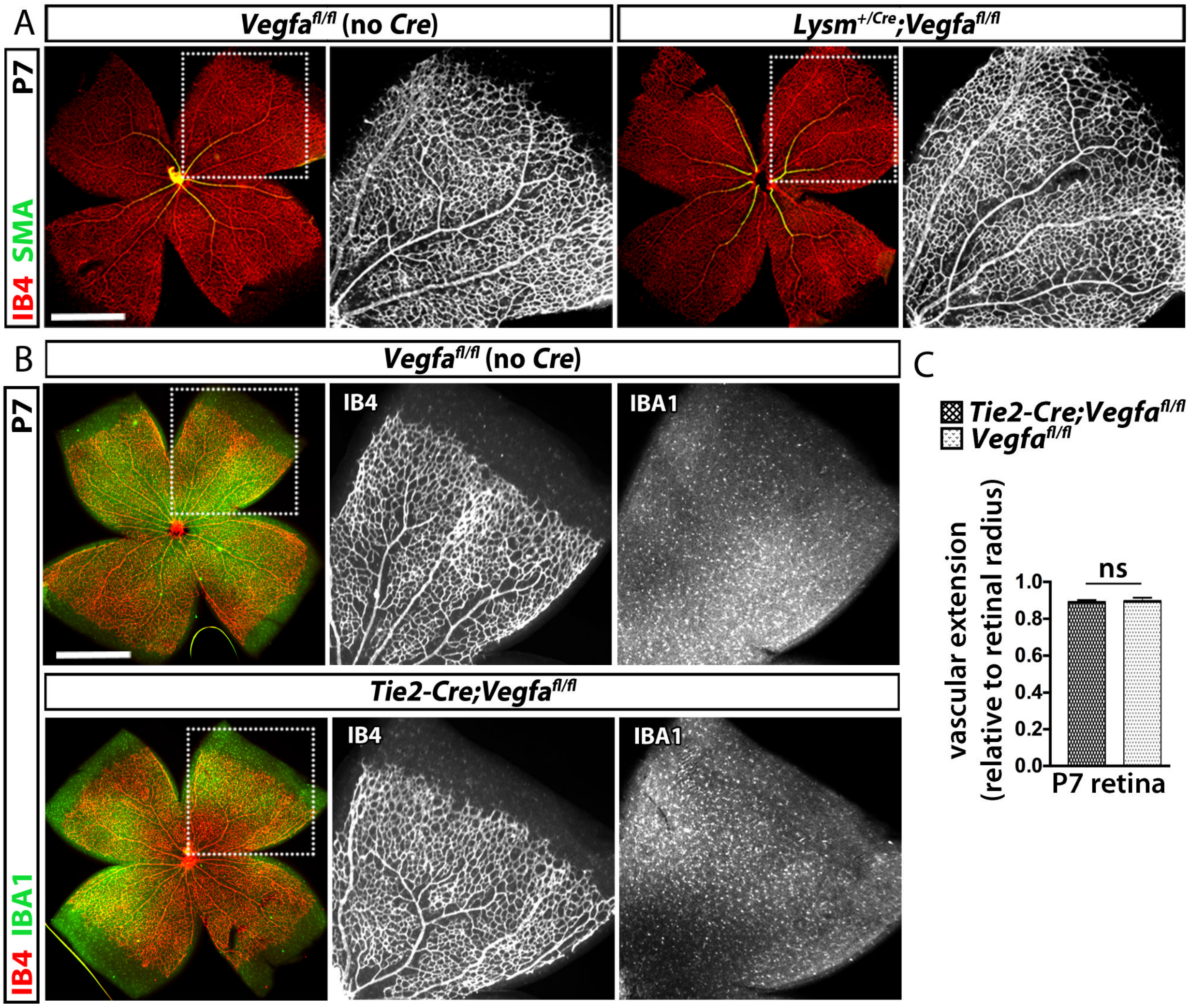


**Supplemental Figure I. Myeloid-derived VEGF is not essential for developmental angiogenesis.** (**A**) Wholemount P7 *Lysm*$^{+/Cre}$;*Vegfa*$^{fl/fl}$ mutant and littermate control retinas, stained for alpha smooth muscle actin (SMA) together with IB4; the black and white panel show higher magnification of the areas indicated with white squares.

(**B,C**) Wholemount P7 retinas (**B**) from *Tie2-Cre;Vegfa*$^{fl/fl}$ mutants and littermate controls stained for IBA1 together with IB4 show a similar density of IBA1$^{+}$ microglia and similar vascular development; the black and white panel show higher magnification of the areas indicated with white squares. There is no significant difference in vascular extension across the retina between both genotypes (**C**); n ≥ 5 mice each, P>0.05, t-test.

Scale bars: 1 mm (**A,B**).

Supplemental Figure II

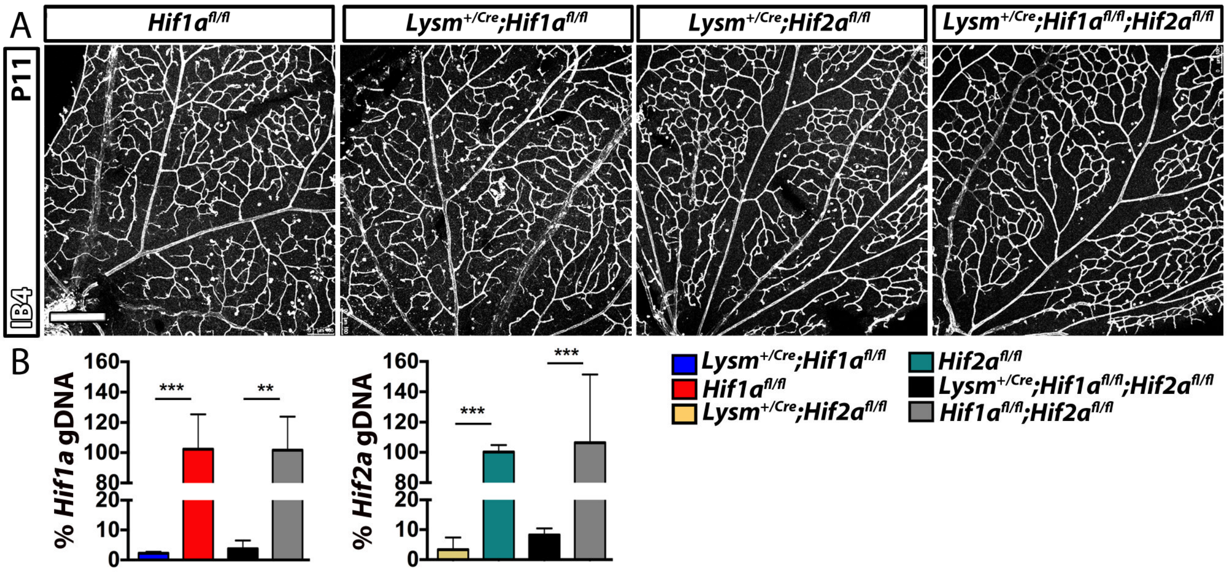


**Supplemental Figure II. Myeloid-derived HIF is not essential for developmental angiogenesis.**
(**A**) IB4 staining of wholemount P11 retinas shows similar vascular development in myeloid *Hif1a*-, *Hif2a*- and double *Hif1a*- *Hif2a*-deficient mice compared to littermate controls.
(**B**) qPCR quantification of *Hif1a* and *Hif2a* genomic DNA levels in *Hif1a*-, *Hif2a*-, double *Hif1a*- *Hif2a*-deficient and control peritoneal neutrophils revealed efficient excision of *Hif1a* and *Hif2a* in *Cre*-expressing cells; mean±SD, n ≥ 3 mice; **P<0.01, ***P<0.001, 1-way ANOVA.
Scale bar: 200 μm (**A**).

Supplemental Figure III

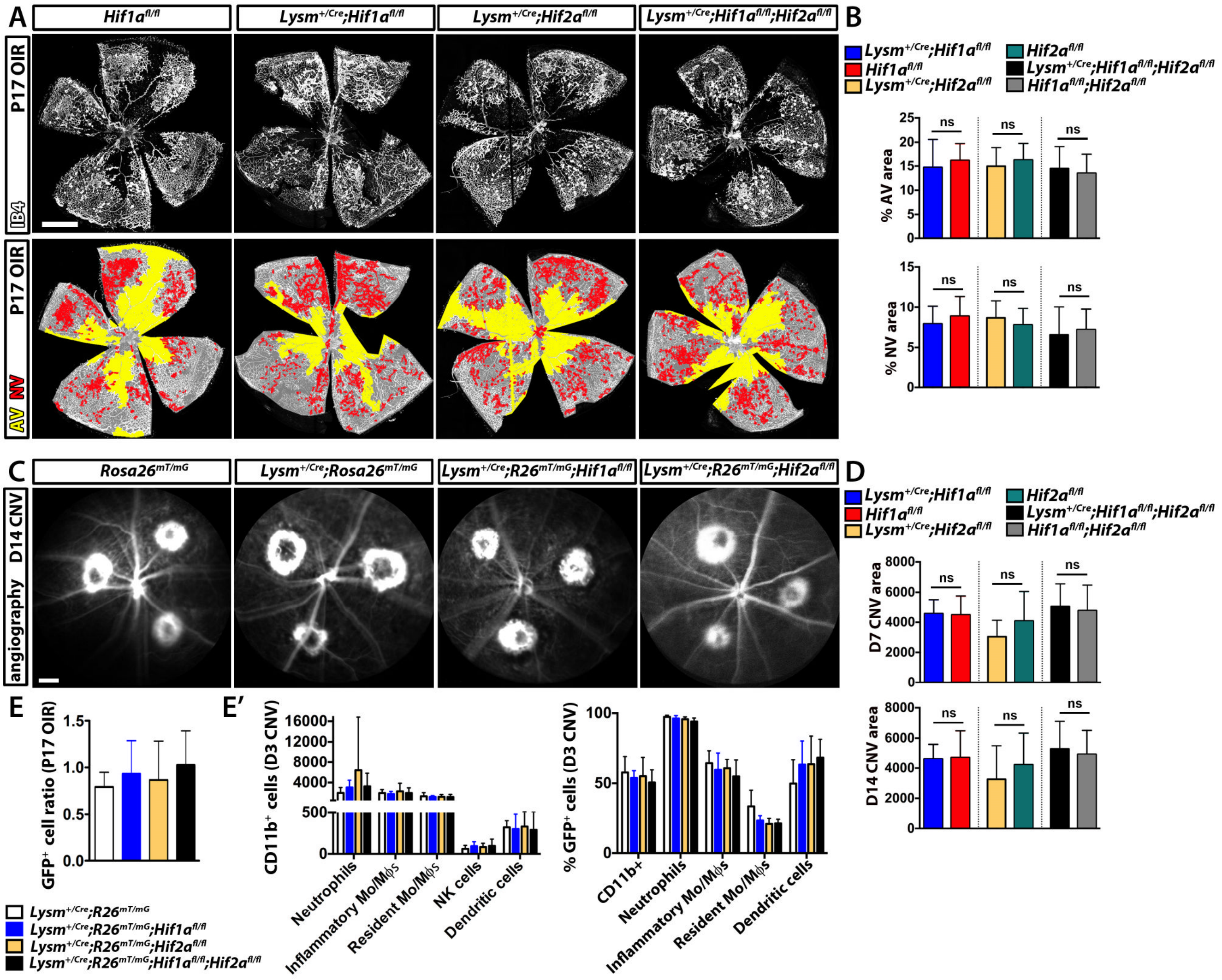


**Supplemental Figure III. *Hif1a* and *Hif2a* expression by myeloid cells is not necessary for ONV**.
(**A,B**) Representative images of retinal flatmounts (**A**) from mice with $Lysm^{+/Cre}$-mediated myeloid targeting of *Hif1a* and/or *Hif2a* and controls, stained with IB4 on P17 in the OIR model; in the bottom panels, total retina, avascular (AV) and neovascular (NV) areas are colour-rendered grey, yellow and red, respectively. (**B**) Quantification of AV and NV areas on P17 in the OIR model for mice with $Lysm^{+/Cre}$-mediated myeloid targeting of *Hif1a* and/or *Hif2a* and controls; mean±SD, n ≥ 10 mice each; P>0.05, 1-way ANOVA.
(**C,D**) Fundus fluorescein angiogram on D14 (**C**) and quantification of average lesion area on D7 and D14 (**D**) after laser injury for mice with $Lysm^{+/Cre}$-mediated myeloid targeting of *Hif1a* and/or *Hif2a* and controls; mean±SD, n ≥ 10 mice each; P>0.05, 1-way ANOVA.
(**E**) Quantification of green fluorescent myeloid cells recruited to NV areas of $Lysm^{+/Cre}$;$Rosa26^{mT/mG}$ retinas on P17 in the OIR model; n ≥ 4 mice each, P>0.05 1-way ANOVA.
(**E'**) Flow cytometric analysis of the choroid/RPE complex on D3 after laser injury of mice with $Lysm^{+/Cre}$-mediated myeloid targeting of *Hif1a* and/or *Hif2a* and controls carrying the $Rosa26^{mT/mG}$ recombination reporter allele, showing the total number of $CD11b^+$ myeloid cells and myeloid cell subpopulations, in addition to $CD11b^+$ NK cells (left), and the percentage of each myeloid subset expressing GFP (right); mean±SD, n ≥ 5 mice each; P>0.05, 1-way ANOVA.
Scale bars: 1 mm (**A**), 2 mm (**C**).
