# Supplementary material for: Myeloid-Derived Vascular Endothelial Growth Factor and Hypoxia-Inducible Factor Are Dispensable for Ocular Neovascularization—Brief Report
Source: Arterioscler Thromb Vasc Biol. 2015 Dec 23;36(1):19–24. doi: 10.1161/ATVBAHA.115.306681 (PMC4684248; doi:10.1161/ATVBAHA.115.306681)
Supplement: Supplementary file 2 [file atv-36-19-s002.pdf]

Materials and Methods

Animals

Animal procedures were conducted with ethical approval under institutional and UK Home Office guidelines. *Lysm*$^{+/Cre}$ mice were crossed to *Hif1a*$^{fl/fl}$, *Hif2a*$^{fl/fl}$ or *Vegfa*$^{fl/fl}$ mice to generate *Lysm*$^{+/Cre}$*;Hif1a*$^{fl/fl}$, *Lysm*$^{+/Cre}$*;Hif2a*$^{fl/fl}$ and *Lysm*$^{+/Cre}$;*Vegfa*$^{fl/fl}$ mutants [1-3]. In some experiments, we used *Tie2-Cre;Vegfa*$^{fl/fl}$ mice [4]. *Vegfa*$^{+/LacZ}$ or floxed *Rosa26*^{Yfp} and *Rosa26*$^{mT/mG}$ were used as reporters of gene expression and *Cre* recombination, respectively [5-7]. OIR was induced as described [8]. Briefly, nursing mothers and their pups were maintained in 75%±3% oxygen from P7 to P12 and returned to room air for 5 days to P17. CNV was induced with a diode laser as described [9]. Fundus angiography with 2% fluorescein was performed on D7 and D14 post-lasering with a scanning laser ophthalmoscope (Heidelberg Spectralis, Germany). Central avascular (AV) and neovascular (NV) areas were measured with ImageJ 1.44i (NIH, USA). Peritoneal neutrophils were isolated after intraperitoneal injection of 30ng lipopolysaccharide (LPS; Sigma, UK).

Histology

Formaldehyde-fixed tissue was labelled with biotinylated isolectin B4 (IB4) (Life Technologies, UK) and antibodies for F4/80 (AbD Serotec, UK), ionized calcium binding adapter molecule-1 (IBA1) (Wako, Japan) or GFP/YFP (MBL International, USA), followed by fluorophore-conjugated streptavidin and appropriate secondary antibodies (Life Technologies, UK). X-gal staining and in situ hybridisation were performed on frozen eye sections [10, 11].

Flow cytometry and sorting

Single-cell suspensions were stained with fluorophore-conjugated antibodies (Biolegend, UK) for CD11b (myeloid cell lineage), CD11c (dendritic cells), Ly6G (neutrophils), NK1.1 (natural killer cells), Ly6C (activation status of macrophages and monocytes). Flow cytometry data were collected on an LSRFortessa (BD Pharmingen, UK) and analysed with FlowJo (Treestar, USA). Influx sorter (BD Pharmingen, UK) was used for cell sorting.

Quantitative PCR (qPCR)

DNA and RNA were isolated using the All-prep kit (Qiagen, UK). RNA was reverse-transcribed with the QuantiTect Reverse Transcription kit (Qiagen, UK) and qPCR performed on an Applied Biosciences 7900HT thermocycler (Life Technologies, UK) using the TaqMan probe-based PerfeCTa® qPCR FastMix® (VWR, UK) or SYBR Green (Life Technologies, UK) and the following oligonucleotides; for genomic PCR, *Vegfa*-F 5'-ACTTCATGGACAGGCTTCGG-3' and *Vegfa*-R 5'-ACATCTGCTGTGCTGTAGGAAG-3'; *Hif1a*-F 5'-TGATGTGGGTGCTGGTGTC-3' and *Hif1a*-R 5'-TTGTGTTGGGGCAGTACTG-3'; *Hif2a* (*Epas1*)-F 5'-GAGAGCAGCTTCTCCTGGAA-3' and *Hif2a* (*Epas1*)-R 5'-TGTAGGCAAGGAAACCAAGG-3'; for RT-PCR, *Vegfa* RT-F 5'-CAGATCATGCGGATCAAACCT-3' and *Vegfa* RT-R 5'-TTGTTCTGTCTTTCTTTGGTCTG-3'; *Hif1a* RT-F 5'-TGGGGATGAAAACATCTGCTTTGGA-3' and *Hif1a* RT-R 5'-GCTTCGCCGAGATCTTGCTGC-3'; *Hif2a* (*Epas1*) RT-F 5'-CCAGGACTAACCCCTCGTTT-3' and *Hif2a* (*Epas1*) RT-R 5'-GGGATTTCTCCTTCCTCAGC-3'; *Actb* RT-F 5'-CACGCCCTTTCTCAATTGTC-3' and *Actb* RT-R 5'-CCAAGGGAGACTCAGCTCAT-3'.

ELISA

VEGF levels were measured with the Duoset ELISA kit (R&D Systems, UK).

References:

1. Clausen BE, Burkhardt C, Reith W, Renkawitz R, Forster I. Conditional gene targeting in macrophages and granulocytes using lysmcre mice. *Transgenic Res*. 1999;8:265-277
2. Cramer T, Yamanishi Y, Clausen BE, Forster I, Pawlinski R, Mackman N, Haase VH, Jaenisch R, Corr M, Nizet V, Firestein GS, Gerber HP, Ferrara N, Johnson RS. Hif-1alpha is essential for myeloid cell-mediated inflammation. *Cell*. 2003;112:645-657
3. Imtiyaz HZ, Williams EP, Hickey MM, Patel SA, Durham AC, Yuan LJ, Hammond R, Gimotty PA, Keith B, Simon MC. Hypoxia-inducible factor 2alpha regulates macrophage function in mouse models of acute and tumor inflammation. *J Clin Invest*. 2010;120:2699-2714
4. Cattin AL, Burden JJ, Van Emmenis L, Mackenzie FE, Hoving JJ, Garcia Calavia N, Guo Y, McLaughlin M, Rosenberg LH, Quereda V, Jamecna D, Napoli I, Parrinello S, Enver T, Ruhrberg C, Lloyd AC. Macrophage-induced blood vessels guide schwann cell-mediated regeneration of peripheral nerves. *Cell*. 2015;162:1127-1139
5. Miquerol L, Gertsenstein M, Harpal K, Rossant J, Nagy A. Multiple developmental roles of vegf suggested by a lacz-tagged allele. *Dev Biol*. 1999;212:307-322
6. Srinivas S, Watanabe T, Lin CS, William CM, Tanabe Y, Jessell TM, Costantini F. Cre reporter strains produced by targeted insertion of eyfp and ecfp into the rosa26 locus. *BMC Dev Biol*. 2001;1:4
7. Muzumdar MD, Tasic B, Miyamichi K, Li L, Luo L. A global double-fluorescent cre reporter mouse. *Genesis*. 2007;45:593-605
8. Smith LE, Wesolowski E, McLellan A, Kostyk SK, D'Amato R, Sullivan R, D'Amore PA. Oxygen-induced retinopathy in the mouse. *Investigative Ophthalmology & Visual Science*. 1994;35:101-111
9. Balaggan KS, Binley K, Esapa M, MacLaren RE, Iqball S, Duran Y, Pearson RA, Kan O, Barker SE, Smith AJ, Bainbridge JW, Naylor S, Ali RR. Eiav vector-mediated delivery of endostatin or angiostatin inhibits angiogenesis and vascular hyperpermeability in experimental cnv. *Gene Ther*. 2006;13:1153-1165
10. Ruhrberg C, Gerhardt H, Golding M, Watson R, Ioannidou S, Fujisawa H, Betsholtz C, Shima DT. Spatially restricted patterning cues provided by heparin-binding vegf-a control blood vessel branching morphogenesis. *Genes & Development*. 2002;16:2684-2698
11. Darland DC, Massingham LJ, Smith SR, Piek E, Saint-Geniez M, D'Amore PA. Pericyte production of cell-associated vegf is differentiation-dependent and is associated with endothelial survival. *Dev Biol*. 2003;264:275-288
